# Dental Adhesive Interfaces Reinforced with Magnetic Nanoparticles: Evaluation and Modeling with Micro-CT versus Optical Microscopy

**DOI:** 10.3390/ma13183908

**Published:** 2020-09-04

**Authors:** Cristian Zaharia, Virgil-Florin Duma, Cosmin Sinescu, Vlad Socoliuc, Izabell Craciunescu, Rodica Paula Turcu, Catalin Nicolae Marin, Anca Tudor, Mihai Rominu, Meda-Lavinia Negrutiu

**Affiliations:** 1 School of Dental Medicine, “Victor Babes” University of Medicine and Pharmacy of Timisoara, 300070 Timisoara, Romania; zack_cri@yahoo.com (C.Z.); atudor@umft.ro (A.T.); mrominu@hotmail.com (M.R.); medanegrutiu@gmail.com (M.-L.N.); 2 3OM Optomechatronics Group, Faculty of Engineering, “Aurel Vlaicu” University of Arad, 310130 Arad, Romania; 3 Doctoral School, Polytechnic University of Timisoara, 300222 Timisoara, Romania; 4 Centre for Fundamental and Advanced Technical Research, Laboratory of Magnetic Fluids, Romanian Academy—Timisoara Branch, 300223 Timisoara, Romania; vsocoliuc@gmail.com; 5 National Institute for Research and Development of Isotopic and Molecular Technologies, 400293 Cluj-Napoca, Romania; izabell.craciunescu@itim-cj.ro (I.C.); rodica.turcu@itim-cj.ro (R.P.T.); 6 Faculty of Physics, West University of Timisoara, 300223 Timisoara, Romania; cnicolaem@gmail.com

**Keywords:** dental adhesive, magnetic nanoparticles, micro-CT, optical microscopy, adhesive layer thickness, modeling, multi-parametric analysis

## Abstract

Dental adhesives are used in a wide range of applications, including to place direct composite restorations in frontal or posterior teeth. One of the most frequent causes for the failure of composite resin restorations is microleakages. The first aim of this work is to introduce a new type of self-etched dental adhesive doped with magnetic nanoparticles (MPs) synthetized in the laboratory. The scope is to produce adhesives with a minimized width/thickness to decrease the risk of microleakages. The second aim is to assess the width/thickness of the adhesive layer in all the characteristic areas of the teeth using both the less precise but most common optical microscopy and the more accurate and volumetric micro-Computed Tomography (CT) investigations. Twenty extracted teeth have been divided into four groups: Group 1 includes ‘blank’ samples with adhesives that are not doped with MPs; Group 2 includes samples with adhesives doped with MPs; Groups 3 and 4 include samples with adhesives doped with MPs that are subjected to an active magnetic field for 5 and 10 min, respectively. Microscopy investigations followed by micro-CT and EDAX are performed on the adhesive. While a rather good agreement is obtained between the microscopy and micro-CT results, the capability of the latter to offer a full volumetric reconstruction of the layer is exploited to analyze the adhesion of the four considered dental materials. Thus, from micro-CT results the graphs of the surface areas as functions of the adhesive layer width are modeled mathematically, as well as the volume of sealants, for each of the four groups. To our knowledge, it is the first time that such a methodology is used. Characteristic parameters are extracted and the ascertainment of the optimal parameter that should be utilized for such assessments is discussed. The study demonstrates the adhesion improvement produced for Groups 3 and 4, where MPs are used. It also concludes that the magnetic field should be applied to the adhesive material for the longest possible exposure time (with a trade-off with the clinical duration of the treatment).

## 1. Introduction

Dental adhesives are used in numerous applications, which include the following: to place direct composite restorations in frontal or lateral teeth; to cement composite, porcelain or metal-reinforced prosthetic restorations (e.g., inlays, onlays, fixed partial dentures, or veneers); to cement indirect prosthetic fixed restorations [1]. They must provide as tight a connection as possible between the composite material used in the restoration of dental cavities (polymers reinforced with different inorganic fillers [2]) and the dental surface. To achieve this, adhesive materials must display low viscosity, substrate wettability, and fluidity [3]. They should also ideally eliminate microleakages, which manifest as the passage of fluids, bacteria, molecules or ions in the connection between the restoration material and the cavity walls [4]. Microleakages are one of the most frequent causes for the failure of composite resin restorations and from their point of view. Adhesives have a similar behaviour at the tooth/restoration material interface, regardless of their type: etch-and-rinse or self-etch [5,6]. A first direction of research in dental adhesives is therefore to develop novel materials, capable to provide a tighter connection between the tooth and the dental restoration material.

A second direction of research in this domain refers to the development and application of different techniques to analyze occurrence of microleakages, including [7,8,9,10,11]: (i) penetration with different dyes, such as fuchsin or methylene blue; (ii) testing of marginal integrity by using penetration with radioactive isotopes, bacteria, or using the pressure of a compressed air source; (iii) electronic and electrochemical microleakage monitoring; (iv) imaging assessments with scanning electron microscopy (SEM) [12], replication and SEM, fluorescent, confocal or stereomicroscopy [13,14,15], as well as optical coherence tomography (OCT) [16,17,18,19,20,21,22].

The two aims of this work address both directions of research above: (1) to develop an improved dental adhesive material that is capable of assuring smaller widths/thicknesses in the tooth/composite material interface; (2) to assess the efficiency of the above materials and procedures using two different imaging methods, optical microscopy (the most common one) and micro-CT (which allows for a complete, volumetric and exact evaluation).

Regarding the first aim above, magnetic nanoparticles (MPs) manufactured in the laboratory are inserted in the adhesive, which is prepared with different procedures to optimize the adhesive distribution in the interface. Nanoparticles [23,24,25,26,27,28,29,30], including MPs are largely used in medicine [31,32,33,34,35]. To our knowledge, we proposed using MPs for the first time in a series of preliminary studies [36,37,38] with the scope of obtaining a tighter connection between the composite material and the dental surface [39,40], compared with a conventional treatment. To demonstrate the effect of the MPs, dental adhesives with or without MPs are used in this study. The former is applied to the surfaces of the teeth by using the conventional method. The latter is applied by rubbing and maintaining the tooth in a magnetic field for certain periods of time to determine the effect of this exposure time to the adhesive widths.

Regarding the second aim of the study, the question one must answer is: how credible are the results provided using microscopy versus those provided using micro-CT? Another aspect to be approached in the study refers to using the quantifiable micro-CT results to model mathematically the adhesion of the various materials obtained, with the scope to compare them, but also to provide a methodology to study this phenomenon.

## 2. Materials and Methods

### 2.1. Study Groups

In this study, 20 teeth with class I and II cavities have been used. The chosen adhesive has been Evetric Bond (Ivoclar Vivadent AG, Schaan, Principality of Liechtenstein), which provides a homogeneous wetting of the dental surfaces. Therefore, a uniform film is produced in the areas where it is applied. The 20 samples have been divided into 4 groups, as follows:

Group 1 includes ‘blank’ samples with an adhesive that is not loaded with MPs.

Group 2 includes samples with adhesives that are doped with MPs.

Group 3 includes samples with adhesive loaded with MPs that are applied in an active magnetic field for 5 min. This time interval has been chosen by considering that the processing time for the preparation of the adhesive in a patient’s mouth should not be longer than a few minutes.

Group 4 includes samples with adhesives loaded with MPs that are applied in an active magnetic field for 10 min, which is an extended time interval for this process, that has been chosen with the aim of assessing the possible effect of a double (although unusually long) magnetization time interval-with regard to the samples of Group 3.

### 2.2. Preparation and Characterization of the MPs

The adhesive materials of the Groups 2 to 4 were filled with *multicore-shell Fe_3_O_4_-SiO_2_ magnetic particles* synthesized by covering with SiO_2_ colloidal stabilized multicore magnetite nanoparticle clusters. The Fe_3_O_4_ nanoparticles were obtained by means of chemical coprecipitation of Iron (II) and Iron (III) chloride (Merck, Kenilworth, NJ, USA, no purity information) with NaOH (Merck, Kenilworth, NJ, USA, no purity information). The ~8 nm diameter Fe3O4 nanoparticles were sterically stabilized with oleic acid (90%, Sigma-Aldrich, St. Louis, MO, USA) [41].

Ferrofluid containing oleic acid monolayer-coated magnetite nanoparticles stably dispersed in toluene, prepared according to the procedure described in [42] were obtained in the Laboratory of Magnetic Fluids of the Romanian Academy–Timisoara Branch. Sodium lauryl sulfate (SLS) as surfactant for clusters preparation and Tetraethyl orthosilicate (TEOS, 98%), absolute ethanol, ammonia solution (25%) for silica coverage were purchased from Sigma-Aldrich, Inc. and used as received without further purification.

The magnetic clusters (MCs) were prepared using the oil in water mini-emulsion method [43,44,45]. Toluene-based ferrofluid (0.5 wt % Fe_3_O_4_) was added to an aqueous solution containing the surfactant (1.795 g). The presence of SLS surfactant molecules resulted in the formation of micelles, where the surfactant molecules organized themselves with the polar end in the water phase and the non-polar end in the oil phase. The as-created droplets contained the MPs dispersed in toluene. To obtain a stable mini-emulsion, the two-phase mixture was homogenized using an ultrasonic finger U.P. 400S, for 2 min. In the second step, the organic phase, toluene, was evaporated under magnetic stirring (500 rpm) at 100 °C in an oil bath. The MCs were subsequently washed with methanol-water mixture (50 mL) to remove any excess of reactants and then re-dispersed in distilled water.

The as prepared MCs were covered with a SiO_2_ shell. The synthesis of magnetic nanocomposites (Fe_3_O_4_-SiO_2_) was performed using the modified Stober method, by hydrolysis of the SiO_2_ precursor, TEOS in the presence of the as prepared MCs [46,47]. In a typical synthesis of Fe_3_O_4_-SiO_2_, an aqueous solution of ethanol (320 mL) and water (80 mL) with a volume ratio of ethanol/water equal to 4:1 was mixed with 0.4 g (1 wt %) of dry magnetic clusters and the as prepared solution was sonicated using an ultrasonic finger for 10 min. Then the solution was mixed with 8 mL of aqueous ammonia solution (25 wt %) and 6 mL TEOES (18 mM), which was added drop wise into the MPs solution under vigorous magnetic stirring. The reaction was kept to room temperature for 1 h and magnetic stirring (500 rpm). When the reaction time was finished, the product, Fe_3_O_4_-SiO_2_ was separated by an external magnet and washed several times with distilled water. The final product was collected and dried at 60 °C for 12 h.

**Figure 1:**
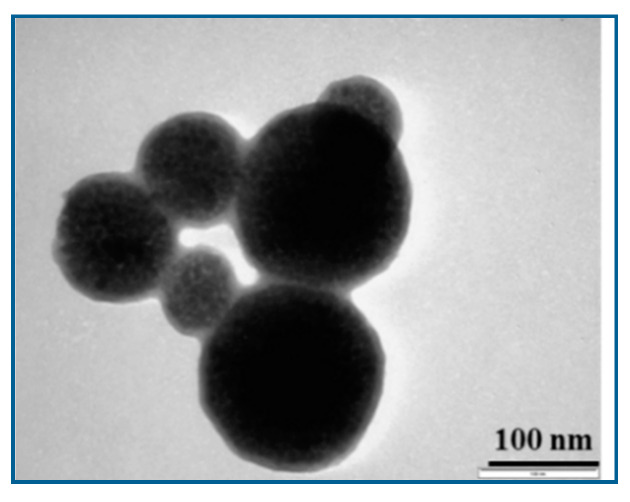
TEM image of manufactured multicore-shell Fe_3_O_4_-SiO_2_ magnetic nanoparticles (MPs).

The MPs were investigated using Transmission Electron Microscopy (TEM), with a JEOL JEM-1010 system (JOEL Ltd., Tokyo, Japan). TEM investigations showed that the MPs have spherical shapes and sizes in the range of 50 to 200 nm—Figure 1. Their core consists of highly packed MPs with a ~8 nm diameter obtained as described above. The thickness of their SiO_2_ shell was evaluated at approximately 20 nm.

The magnetization properties of the MPs were measured at room temperature in the field range of 0 to 1000 kA/m, using a vibrating sample magnetometer (VSM 880, DMS/ADE Technologies, USA). The obtained MPs have superparamagnetic behavior, with a 21 emu/g saturation magnetization.

An EDAX analysis was also performed, using a SEM high vacuum FEI Quanta 250 system and a secondary Everhard–Thomley electron detector. EDAX analysis parameters were HV mode, 15 kV, ETD, EDAX, spot 5 and WD 5 mm.

### 2.3. Preparation of Samples

**Figure 2:**
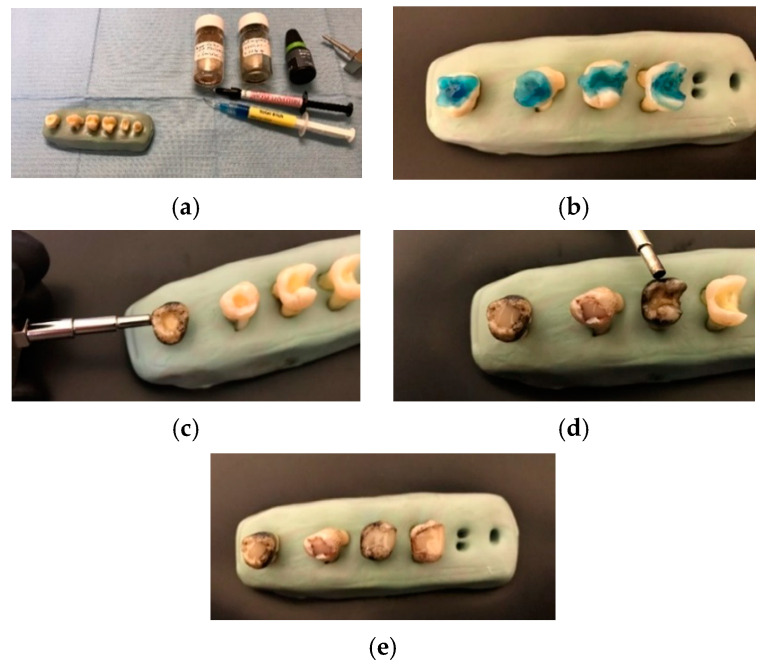
Processing of samples (examples): (**a**) study armamentarium and (**b**) etchant gel applied for 15 s on the dentin and for 30 s on the enamel; (**c**,**d**) application of the magnetic field on the dental adhesive (for 5 or 10 min, for Groups 3 and 4, respectively); (**e**) final samples with MPs fillings.

Several methods were used to prepare the samples of each group from different points of view, as detailed in the following. For Group 1, five ‘blank’ samples were created using the usual protocol for filling a tooth that has a cavity on its surface, namely: dentinal demineralization for 15 s; enamel demineralization for 30 s with BlueEtch demineralizing gel (Cerkamed, Stalowa Wola, Poland)—Figure 2b; washing of the demineralizing gel and cavity drying; application of the non-filled MPs adhesive and its dispersion with air; polymerization for 20 s; filling of the cavity with a radio-opaque flowable composite, and then polymerization for 40 s.

For Groups 2 to 4, the adhesive was mixed with the MPs used in the study, as presented in Section 2.1. For the five samples of Group 2, the dental adhesive loaded with MPs has been applied conventionally. For each of the five samples (loaded with MPs) of Groups 3 and 4 a magnetic field was applied for 5 and 10 min, respectively, using a permanent magnet applicator, as shown in Figure 2c,d. After the application of the MPs-loaded adhesive, the cavities were filled with a fluid composite material, as shown in Figure 2e.

**Figure 3:**
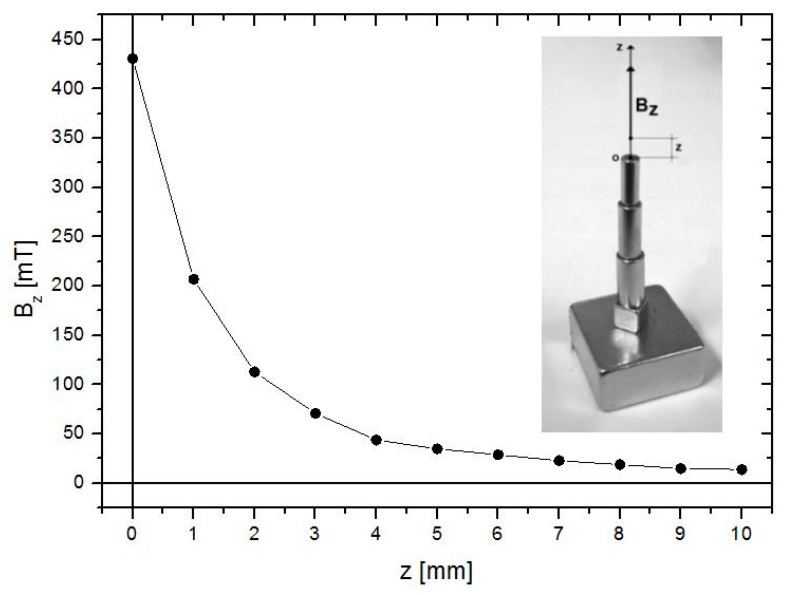
The induction of the magnetic field as a function of the distance *z* from the applicator tip.

One must highlight that in the second step of this procedure, the permanent magnet applicator must be kept as close as possible to the adhesive doped with MPs, as the magnetic field induction as a function of the distance *z* from the applicator tip drops significantly, as presented in Figure 3.

### 2.4. Optical Microscopy and Micro-CT Investigations

For the first type of investigation, after slicing the samples, their areas were evaluated using an A377 optical microscope (Electromann SA, Pretoria, South Africa) with a magnification of 20× to 800×, a 2MPX CMOS sensor, and a manual focus of up to 40 mm.

For the second type of investigation, after the scanning piece was positioned on the worktop of a Nikon XTH-450 micro-CT system (Nikon Corporation, Tokyo, Japan), the scanning settings were made, and then the actual scanning, reconstruction and interpretation of the results were performed. The micro-CT scanning was carried out around the center of rotation by rotating the samples with 360 degrees; 1000 projections were captured for each sample. They were imported into Vg Studio Max (Volume Graphics, Heidelberg, Germany), and 3D reconstructions were obtained to allow for the analysis of the adhesive interface on the dental surfaces.

## 3. Results

### 3.1. Optical Microscopy Analysis

**Figure 4:**
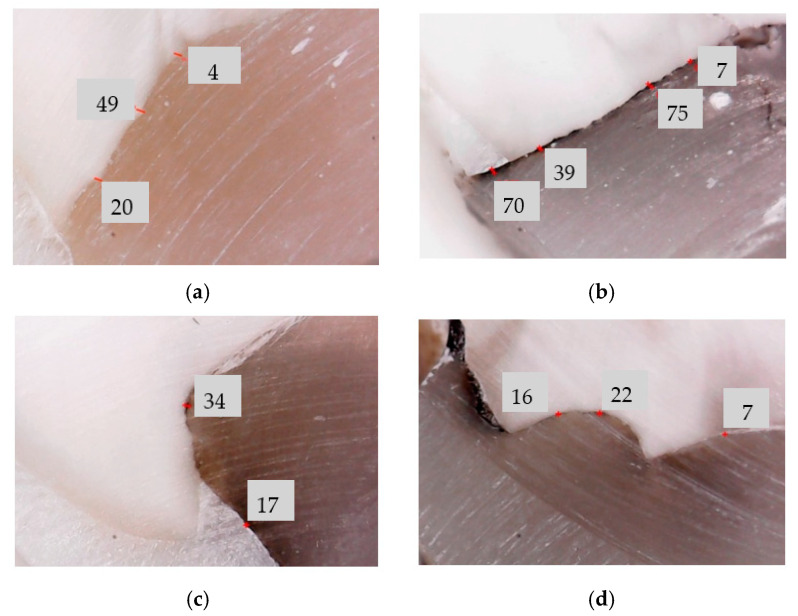
Example of a microscopy analysis of the width/thickness of the dental adhesive layer on a sample: (**a**) without MPs (from Group 1); (**b**) with MPs (from Group 2); (**c**) with MPs applied in a magnetic field for 5 min (from Group 3) and (**d**) for 10 min (from Group 4). The measured adhesive layer widths are provided in μm.

Examples of this initial investigation, which is the most common imaging method used to assess the width/thickness of the adhesive layers are shown in Figure 4 for a sample from each of the four considered groups. Direct measurements were performed on each of the sectioned samples, in 10 characteristic points on each of the three surfaces of the teeth, i.e., on the vestibular, oral, and pulpal interfaces. The average values obtained for each sample are given in Table 1.

The microscopy analysis yielded the following results: (i) for Group 1, the adhesive layer width/thickness (*w*) ranges from 0.021 to 0.29 mm; (ii) for Group 2, *w* ranges from 0.016 to 0.31 mm; (iii) for Group 3, *w* decreased to values between 0.013 and 0.19 mm; for Group 4, the minimum values of *w* were found, from 0.011 to 0.039 mm.

### 3.2. Micro-CT Analysis

The width/thickness of the adhesive layer obtained with this method was roughly between 0.018 and 0.40 mm (Table 1). One can remark that the three areas analyzed using micro-CT generated different results.

Thus, in the case of samples from Group 1 (using non-loaded dental adhesives), the width *w* ranges between 0.02 and 0.4 mm. For samples from Group 2 (adhesive loaded with MPs), *w* ranges between 0.017 and 0.4 mm. For Group 3 samples (adhesives loaded with MPs applied in the magnetic field for 5 min), *w* ranges between 0.013 and 0.31 mm. The minimum width of the adhesive was found, as with microscopy results, in the Group 4 samples (for which the magnetic field was applied to the adhesives for 10 min), namely between 0.011 and 0.032 mm (Table 1).

The statistical analysis of the micro-CT, as well as the of microscopy results of the adhesive layer widths *w* in all three areas of the tooth (oral, vestibular and pulpal) and for all the four considered groups are presented in Table 2 and Table 3. Regarding the limited number of samples in each study group, we verified that it is sufficient by performing a G Power test, with a “Laplace” Parent Distribution, 80% power and 1 as an allocation ratio.

**Figure 5:**
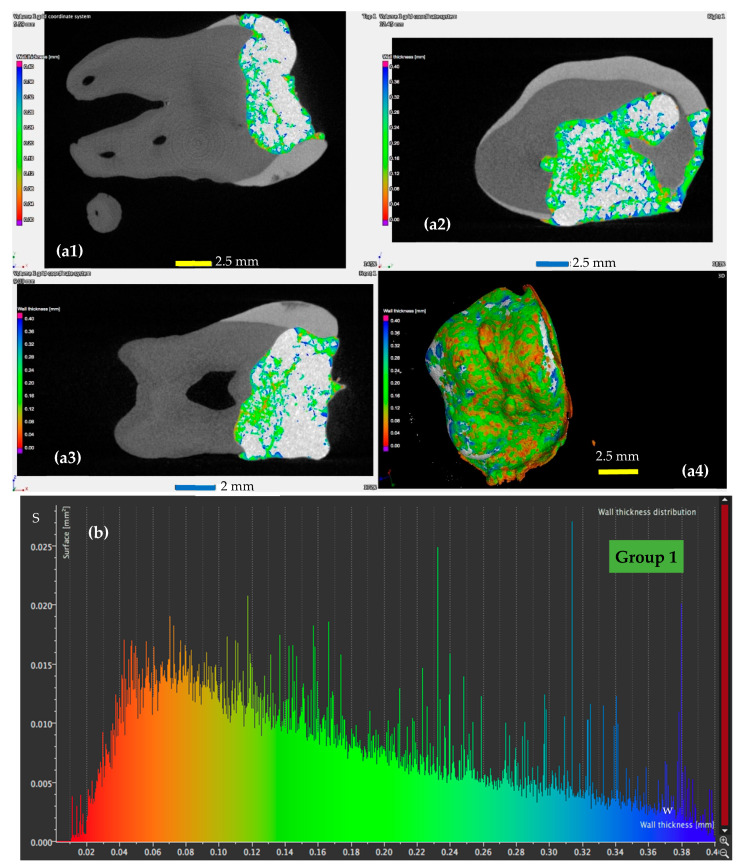
(**a**) Micro-CT investigation on the vestibular (**a1**), oral (**a2**) and pulpal (**a3**) interfaces of a Group 1 sample, with (**a4**) a 3D reconstruction of the dental layer; (**b**) area *S* of the surface of the dental adhesive layer as a function of the width/thickness *w*—example for a Group 1 sample.

**Figure 6:**
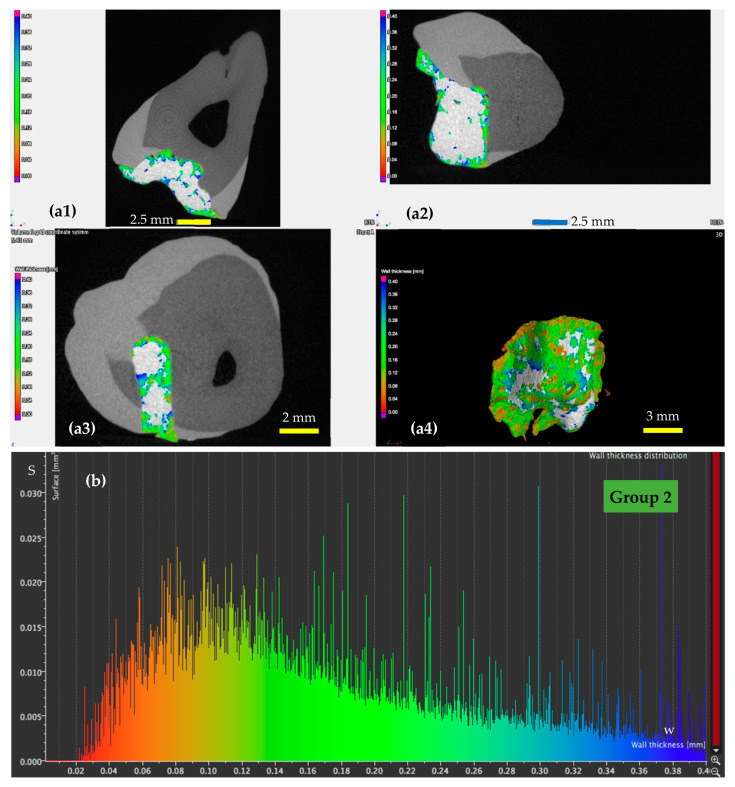
(**a**) Micro-CT investigation on the vestibular (**a1**), oral (**a2**), and pulpal (**a3**) interfaces of a Group 2 sample, with (**a4**) a 3D reconstruction of the dental layer; (**b**) area *S* of the surface of the dental adhesive layer as a function of the width/thickness *w*—example for a Group 2 sample.

**Figure 7:**
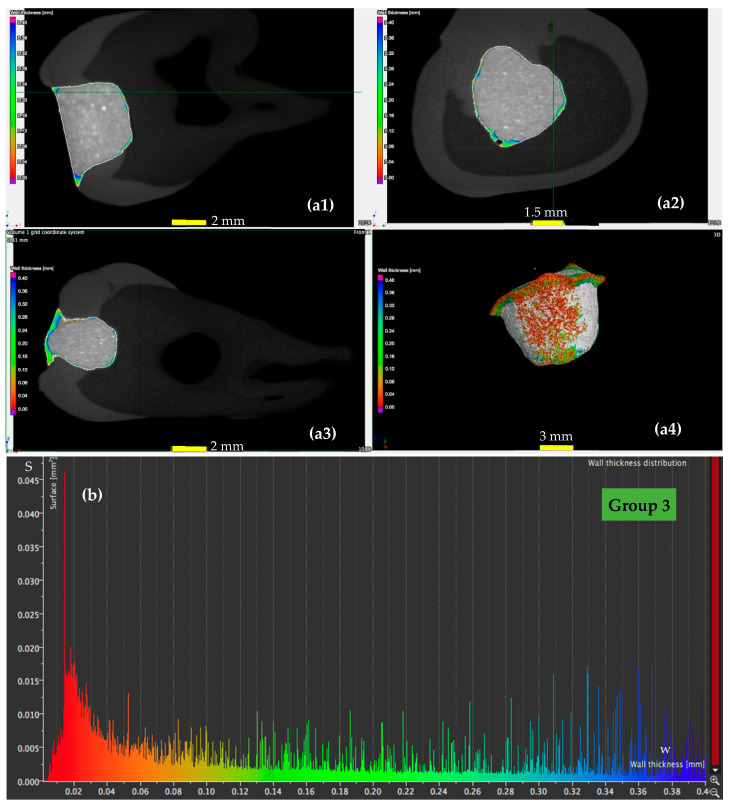
(**a**) Micro-CT investigation on the vestibular (**a1**), oral (**a2**), and pulpal (**a3**) interfaces of a Group 3 sample, with (**a4**) a 3D reconstruction of the dental layer; (**b**) area *S* of the surface of the dental adhesive layer as a function of the width/thickness *w*—example for a Group 3 sample.

**Figure 8:**
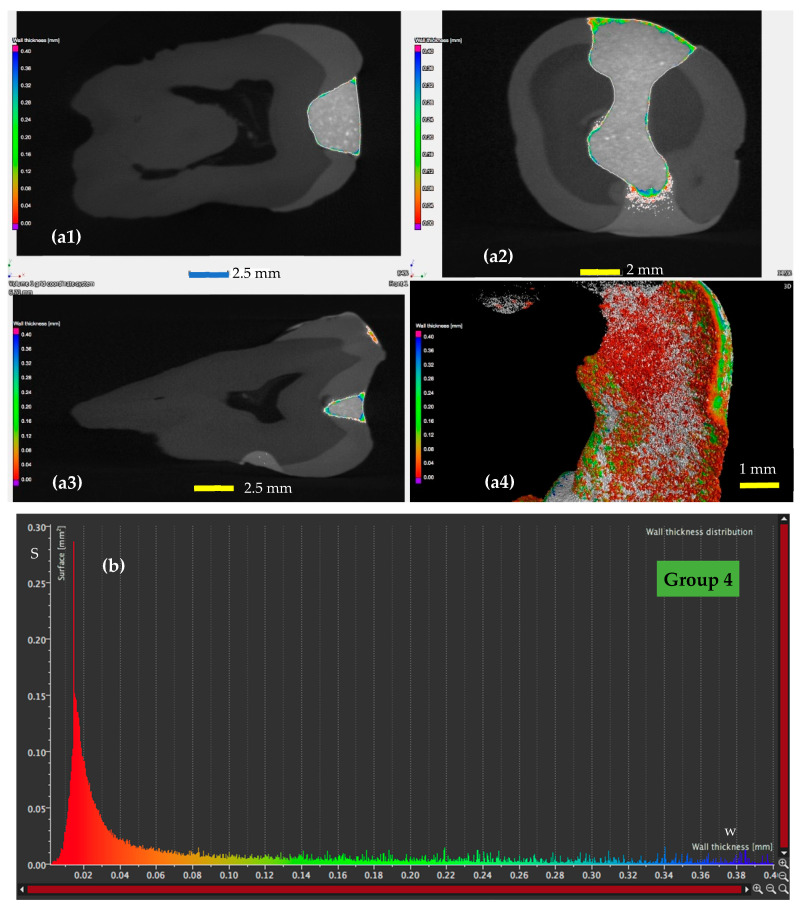
(**a**) Micro-CT investigation on the vestibular (**a1**), oral (**a2**), and pulpal (**a3**) interfaces of a Group 4 sample, with (**a4**) a 3D reconstruction of the dental layer; (**b**) area *S* of the surface of the dental adhesive layer as a function of the width/thickness *w*—for a Group 4 sample.

A detailed analysis using micro-CT is presented for a sample from each of the four groups in Figure 5, Figure 6, Figure 7 and Figure 8, with the remark that the results are similar from each of the five samples in each group. The results for all the samples of each group, as well as their statistics are presented and discussed in Section 4. From the micro-CT results in Figure 5, Figure 6, Figure 7 and Figure 8, the range of values of the width *w* of the adhesive layer is as follows:

(i) For the control teeth of Group 1 (Figure 5), on the surface of which the dental adhesive was applied conventionally showed surfaces with *w* ranging from 0.01 to 0.4 mm. The largest area of the adhesive has a width of around 0.1 mm.
(ii) For the samples of Group 2 (Figure 6), in which the dental adhesive was loaded with MPs in the absence of a magnetic field, the same range of *w* can be observed. A slight decrease of the areas of the surfaces *w* can be observed, which is predominant around a width *w* of 0.08 mm.
(iii) For the samples of Group 3 (Figure 7), loaded with MPs on which a magnetic field was applied for 5 min, the areas of the adhesive with an increased width are getting much lower. Areas with adhesive thicknesses of 0.02 to 0.03 mm are in this case predominant.
(iv) For the samples of Group 4 (Figure 8), loaded with MPs on which a magnetic field was applied for 10 min, the width of the adhesive layer decreases for the largest part of the considered areas. Thus, areas with adhesive thicknesses of 0.01 to 0.025 mm are predominant, while the area peaks that are still present in Figure 7 (for Group 3) do not appear anymore. The areas of the adhesive with increased width (towards the same peak value of area of 0.015 mm^2^) are very low.

These remarks are qualitative, but the graphs obtained in Figure 5, Figure 6, Figure 7 and Figure 8 also allow for a quantitative analysis, as performed in Section 4. This is another advantage of applying micro-CT compared to microscopy, besides providing exact results for the entire volume of the dental adhesive layer.

The results of the micro-CT analysis, considering all the results obtained for all samples of each group are presented in Table 4, Table 5 and Table 6. A comparative look between the different groups has been considered to allow for the discussion of the results performed in Section 4.

### 3.3. EDAX Analysis

**Figure 9:**
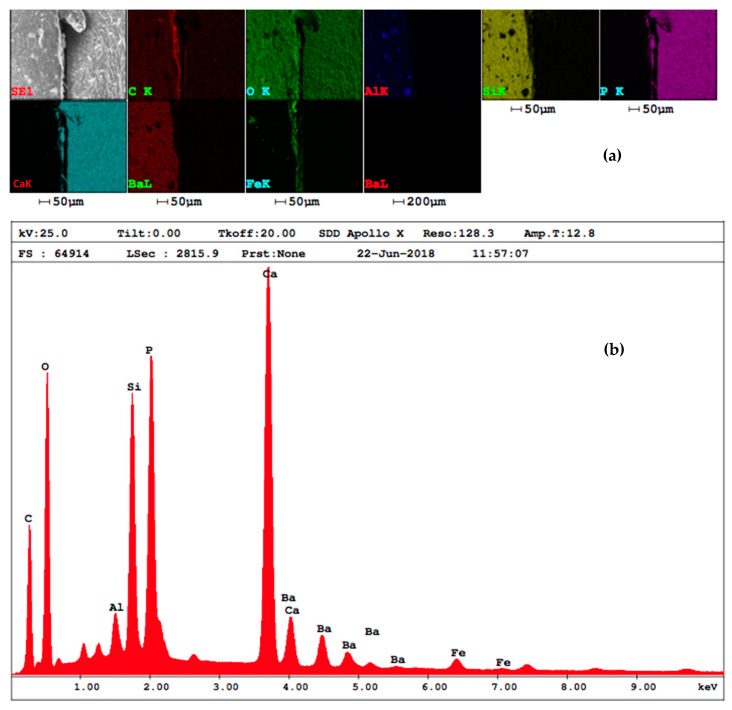
(**a**) Chemical components identified in the dental adhesive layer doped with MPs and (**b**) their diagram.

Magnetic ferrite nanoparticles have been highlighted in the adhesive layer using the EDAX analysis, as shown in Figure 9a. The diagram of the chemical components of the adhesive material doped with MPs has been presented in Figure 9b.

## 4. Discussion

### 4.1. Development of the Novel Adhesive Material

Numerous studies are focused nowadays on the development of novel adhesive materials [48]. Issues addressed include their cytotoxicity [49], strengthening the tooth/composite resin bond [50,51,52,53], and increasing adhesives’ stability [54] and durability [55], as well as improving their performances by adding nanoparticles in their composition [52,56,57,58]. The latter direction of research has been the scope of this work as well.

This effort is justified by the positive impact adhesives have in dental clinical practice, for minimal invasiveness, preserving as much as possible the patient’s dental hard tissue structure and achieving aesthetic restorations, the latter using composite resins [2].

The stress and contraction that appear during their polymerization can influence the width/thickness of the adhesive layer, hence the necessity to improve the clinical performances of these adhesives. The scope, as followed in this study as well, is to provide a tight tooth/composite material interface, therefore a small width and homogenous adhesive layer. Failing to achieve this leads to microleakages and ultimately to secondary cavities [59]. Obtaining new, improved adhesives, as demonstrated by using MPs-loaded materials in this work can have a positive impact on durability and bond strength. This approach comes in the context of numerous studies focused on various dental adhesives systems [55,60,61,62]. While the present study has demonstrated improvements on the adhesive capability of magnetic MPs-loaded materials, future work is required to determine their bond strength and cytotoxicity [63,64].

In the following, to discuss the effect of including MPs in the dental sealants and exposing them for certain periods of time to a magnetic field, one must first make a comparison between the results obtained with the two considered imaging methods (that should validate each other). Then, the width/thickness results of the tooth/dental composite resin interface can be analyzed for the four considered groups to complete the proposed assessment. Different methods can also be explored in this respect.

### 4.2. Comparison between Optical Microscopy and Micro-CT Results

Optical microscopy is widely used in restorative dentistry assessments, from the preparation of root canals for endodontic treatments to the adhesion of teeth through composite resins, or to prosthetic restorations manufactured in dental laboratories. Thus, stereomicroscopy has demonstrated the presence of microleakage areas, predominantly for composite restorations manufactured using direct techniques, compared to prosthetic restorations made in a dental laboratory [13,14,15]. Other studies have been focused on class V cavities [65], the microleakage of root-end filling materials in endodontics [66,67], and techniques to improve the microleakage qualities of cements [68].

In research this common technology has limitations because it cannot provide 3D views of the considered surfaces. A question also arises regarding the accuracy of its results, as they may differ significantly by considering different positions of the sectioning planes, as observed in Figure 4. The reason for these variances is given by the non-uniform thickness of the adhesive layer, as concluded from the microscopy results of this study as well (Table 1).

Micro-CT can be used to investigate completely (over the entire adhesive volume) and accurately the width of the dental adhesive layer, as also demonstrated in this work. It can detect microleakage areas generated by gaps between the tooth interface and the composite filling material, as approached in numerous studies [69,70,71]. Nowadays, a requirement of dental research is to analyze samples in a non-destructive manner. In this respect, micro-CT allows for the analysis of the dental adhesive layer by producing 3D images to discover areas where the width of the adhesive is non-uniform, therefore where microleakages may appear, as used in the present study, as well.

Regarding the assessment of the efficiency of the materials and procedures introduced in this work, microscopy is the most used method for dental adhesive layers measurements, but it has the disadvantage of making evaluations only in the sectioning plane. If another sectioning plane is chosen, the results are clearly different, as remarked above. In contrast, micro-CT allows for a complete (i.e., 3D/volumetric) and exact assessment. The question one must answer is: how credible are the results provided using the common method (i.e., microscopy) versus those provided by the most accurate one (i.e., micro-CT)?

Differences between measurements of the adhesive layer using the two methods can be remarked from Table 1. To assess the concordance of the results obtained with microscopy and micro-CT, a statistical comparison is made in Table 2 and Table 3, using the Wilcoxon Signed Ranks Test, for all four groups. Significant (s) differences have been obtained only for the oral area measurements, with higher values obtained using microscopy. In the vestibular and pulpal areas, the values obtained with microscopy are lower, but statistically insignificant (is).

Thus, one may conclude that despite the variances pointed out for microscopy assessments, their results can be considered rather accurate as concerns practical/clinical purposes. However, exact values, and most of all, results that allow for quantitative assessments can be obtained only using micro-CT (Figure 5, Figure 6, Figure 7 and Figure 8), and we shall demonstrate their use in the following, exploiting micro-CT’s advantage in offering a complete view of the adhesive layer. To our knowledge, such a mathematical modeling of the adhesive layer has not yet been done. However, it is useful to allow the comparison of various adhesive materials, providing a novel methodology to study this phenomenon. Different functions and parameters that can be obtained from such an analysis can be compared to fulfill this aim.

Another imaging technique that can be considered for such assessments is OCT [16,17,18,19,20,21,22]. This IR laser-based low-coherence tomography technique allows for the analysis of the surface of interfaces between the restoration material and the dental structures [20]. It shows that there are areas where the interface is optimal, but other areas present microleakages; such results have been validated with micro-CT investigations, in one of our initial studies on this topic [22]. OCT investigations of MP-doped adhesives are the subject of future work. OCT can be applied instead of (or in parallel with) micro-CT for the volumetric reconstruction. A drawback in this respect is the limited penetration depth of OCT, which would impose retrieving a mosaic image [72] (i.e., one for each of the three areas of the interface). In addition, OCT is less performant than micro-CT in terms of resolution, but it is worth considering because of its demonstrated capability to perform non-invasive, real-time and in vivo investigations in the oral cavity using handheld scanning probes [73].

As a general remark, for all imaging methods, to have good results in the investigation of different adhesives, it is necessary to increase the reflectivity, and therefore the accuracy in differentiating the adhesive layer from air inclusions. Including MPs in the (dental) material is a good measure to achieve this scope.

### 4.3. EDAX Analysis

Considering that the adhesive utilized in this study has almost the same refraction index as air, the interface between the tooth structure and the filling material could include both adhesive and air inclusions. For this reason, a SEM with EDAX has been done on the interface to evaluate the adhesive layering. Thus, Figure 9a demonstrates the presence of MPs, while the diagram of the chemical components of the interface in Figure 9b corresponds to the adhesive material doped with MPs; therefore, the presence of air inclusions has been ruled out.

### 4.4. Effect of MPs Inclusion in the Adhesives: Comparison between the Four Groups Using Micro-CT

The first aim of this work has been to develop novel dental adhesives filled with MPs manufactured in the laboratory following the technique described in detail in Section 2. Nanoparticles are widely used in biomedical applications, including orthopaedic (to reinforce composite materials for the replacement of damaged cartilage, meniscus, etc.) [23,24,25], drug delivery [26,27,28], viral sensing [29] or dental materials applications [30]. MPs are a category of nanoparticles of special interest that covers numerous such applications, including diagnosis, biosensing, tissue and immune therapy, regenerative medicine and dentistry [31,32,33,34]. MPs used in biomedical applications are manufactured from certain materials: metals (Fe or Co), different alloys (such as Au/Fe), oxides (magnetite or maghemite), as well as different types of ferrites. Magnetite has been the material of choice for the MPs manufactured for this study (and in general for the dental materials applications considered in our group) because of its ferromagnetic properties, high stability, easy preparation process to a certain size, good shape and porosity and no swelling variations. They also have a low toxicity that makes them suitable for biomedical materials [31,35].

To our knowledge, we have proposed using MPs for the first time in a series of preliminary studies [36,37,38] with the scope of obtaining a tighter connection between the composite material and the dental surface [39,40] and therefore a smaller thickness of the dental adhesive compared with a conventional treatment. To demonstrate the effect of the MPs, dental adhesives with or without MPs have been utilized in this study.

Samples with adhesives loaded with MPs (Group 2), with adhesives with MPs and a magnetic field applied for two different time intervals (Groups 3 and 4) were considered in contrast with the control Group 1 (with samples prepared with a conventional method). As discussed in the previous subsection, micro-CT is commonly used to evaluate micro-infiltrations for dentures and denture sealants, as well as adhesives restorations [20]. From 3D micro-CT assessments, the results regarding the width/thickness of the adhesive layer obtained in the present study have been shown to be in good agreement with the values given in the literature, of about 0.02 mm [21,22,23]—for Group 1, and even for Group 2.

A comparison has been made between the results of different groups in term of the adhesive layer width *w,* using the Mann-Whitney U Test. As it can be seen from Table 4, differences between widths obtained with a conventional technique and with an adhesive with MPs are not significant (s) for all three areas, as obtained in a preliminary study as well [29]. This has imposed looking for a method to take advantage of the potential of the MPs, i.e., subjecting them to a magnetic field. *Their composition has been chosen and they have been specifically manufactured for this purpose.*

In contrast to the above, the width values of Group 3 are significantly (s) lower than those of Group 1 in two areas (vestibular and oral), and insignificantly (is) different in the pulpal area, as shown in Table 5. The same conclusion can be reached when comparing the values obtained for Groups 3 and 2 (not shown here). Therefore, applying a magnetic field even for shorter periods of time improves the adhesion, by minimizing the width of the adhesive material layers.

Furthermore, it can be determined that the width values of Group 4 are significantly lower in all three areas than those of all the other three groups. In Table 6 the final, most relevant comparison, i.e., between Groups 3 and 4 is presented, with a significant (s) decrease of the layer width for the latter in all areas. The differences between Groups 4 and 2 (not shown here) are even larger.

One may thus conclude thus that increasing the time of applying the magnetic field as much as possible (without affecting the patient’s comfort, though) is beneficial for the quality of the dental work, lowering the risk of microleakages.

Future work in our groups includes the encapsulation of the MPs to improve the aesthetic effect of the adhesive. This also imposes the study of the resulting material to characterize its mechanical properties and behavior from the point of view of microleakages.

### 4.5. Mathematical Modeling of the Micro-CT Results

**Figure 10:**
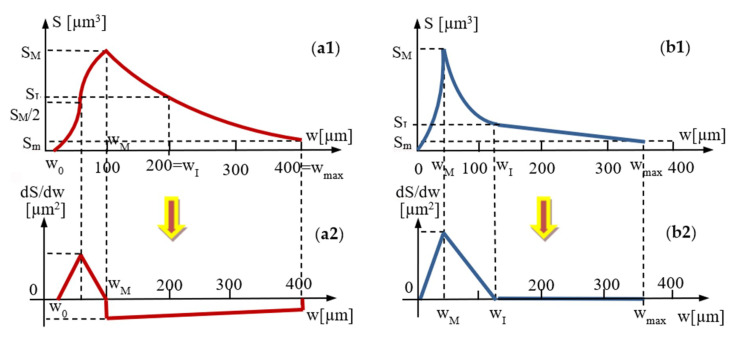
Mathematical modelling of the area *S* of the dental sealant surfaces as functions of their thickness/width *w* for (**a**) Groups 1 and 2—from Figure 5b and Figure 6b, as well as for (**b**) Groups 3 and 4—from Figure 7b and Figure 8b. The *S*(*w*) functions deduced in Appendix A for Groups 1 and 2 and in Appendix B for Groups 3 and 4 are represented with their graphs (1) and gradients (2).

Using the micro-CT diagrams in Figure 5b, Figure 6b, Figure 7b and Figure 8b, the graphs of the surface area *S* of the dental adhesive layer as a function of its width/thickness *w* can be obtained. In Figure 10(a1,b1) these graphs are pointed out—the former for Groups 1 and 2 and the latter for Groups 3 and 4, considering the similar shapes (although with different parameters) of Groups 1 and 2 diagrams, as well as Groups 3 and 4 diagrams, respectively.

Based on these graphs and considering the simplest equations to approximate them on portions (i.e., linear and parabolic), the *S*(*w*) functions are deduced in Appendix A for Groups 1 and 2, and in Appendix B for Groups 3 and 4. These functions are provided, on specific width intervals, in Table 7 and Table 8, respectively.

The gradients of the functions are obtained as well, and they are represented in Figure 10(a2) and Figure 10(b2), respectively. A higher gradient (corresponding to the peak in Figure 8b and Figure 9b) was observed, as well as an approximately constant slope of the *S*(*w*) function for Groups 3 and 4, but this is less relevant in this discussion. However, it shows a tendency of obtaining larger areas for interface surfaces with smaller widths, and much lower areas for surfaces with larger widths. It also indicates the drop in these values when magnetization is applied.

While such conclusions can be extracted from the diagrams in Figure 5b, Figure 6b, Figure 7b and Figure 8b, (without the need to obtain analytical expressions for the corresponding functions), using the functions deduced in Table 7 and Table 8, one can obtain a synthetic, quantitative parameter to characterize adhesion of the different materials. Thus, the most relevant aspect is that, using the *S*(*w*) functions (each one defined on its specific intervals), one may thus obtain *the volume of adhesive material in the interface*:

(1) $$V=\int_{0}^{w_{max}}S\left(w\right)\cdot dw\;\left[{\mathrm{mm}}^{3}\right]$$

This synthetic parameter has been deduced here with this analytic method for the first time to our knowledge. It allows to make a rigorous and simple comparison between the capability of different materials to provide an as tight as possible adhesion, as demonstrated following its values in Table 7 and Table 8. One can thus conclude the decrease in volume of the entire adhesive from the considered Group 1 to the Group 4 sample. This also confirms the results obtained from analyzing and comparing statistically (considering all samples from) each group in Table 4, Table 5 and Table 6. Thus, the two approaches may validate each other.

This methodology can be applied for the micro-CT study of adhesive materials prepared in different ways, as envisaged in our future studies, as well. Other methods, such as OCT, can also benefit from such a quantitative assessment.

## 5. Conclusions

The study introduced and analyzed a novel dental adhesive material loaded with MPs prepared and characterized in the laboratory. We demonstrated that including MPs allows for a significant decrease of the width/thickness of the adhesive layer because a magnetic field can influence them due to their content in Fe. This decrease in width impacts positively the microleakage risk at the dental interface. The best results in terms of the adhesive layer are obtained for adhesives loaded with MPs on which a magnetic field is applied for as long as possible. Therefore, this time interval should be extended as much as possible—while still preserving the patient’s comfort.

A rather good agreement of the results has been obtained with the two considered measuring methods, (the most common) optical microscopy and micro-CT. This is an important conclusion, as microscopy is more available to users. However, in this reciprocal validation, more accurate results have been obtained using micro-CT, which has allowed for a rigorous comparison of the results of the four groups in terms of the width of the dental adhesive layer. A mathematical modeling was also made possible, for the areas of the surfaces of adhesive layer as a function of their widths. Using these obtained functions, volumes of adhesive material for each considered sample were calculated and compared. A novel assessment methodology, quantitative and accurate, which considers the entire volumetric distribution of adhesive has been thus obtained-for the first time to our knowledge.

Future work in our groups includes the encapsulation of the MPs to improve the aesthetic effect of the adhesive. This also imposes the study of the resulting material to characterize its mechanical properties and behavior from the point of view of microleakages.

## Figures and Tables

**Table 1:** Adhesive layer width/thickness *w* (μm) measured with (i) microscopy and (ii) micro-CT in all three areas of the teeth (oral, vestibular, and pulpal).

Group	Sample	Average Values of the Width/Thickness of the Dental Adhesive Layer, *w* (μm)					
		Vestibular Interface		Oral Interface		Pulpal Interface	
		Microscopy	*Micro-CT*	Microscopy	*Micro-CT*	Microscopy	*Micro-CT*
1	1	31	*20*	230	*210*	290	*180*
	2	90	*90*	47	*71*	170	*100*
	3	240	*31*	210	*47*	90	*170*
	4	21	*400*	63	*220*	60	*91*
	5	50	*72*	37	*61*	59	*20*
2	1	21	*17*	71	*21*	42	*92*
	2	120	*110*	170	*73*	30	*64*
	3	53	*400*	160	*56*	16	*270*
	4	67	*29*	31	*46*	70	*23*
	5	59	*190*	130	*210*	310	*77*
3	1	37	*13*	47	*19*	41	*22*
	2	50	*18*	22	*24*	90	*98*
	3	29	*32*	30	*38*	54	*29*
	4	180	*88*	40	*91*	61	*100*
	5	13	*36*	190	*66*	170	*310*
4	1	29	*17*	40	*23*	31	*20*
	2	21	*21*	12	*16*	33	*32*
	3	31	*30*	37	*21*	17	*11*
	4	11	*21*	39	*11*	21	*15*
	5	37	*14*	29	*24*	22	*27*

**Table 2:** Comparison between microscopy and micro-CT results (the adhesive layer widths *w*) in all three areas (oral, vestibular and pulpal), and for all four groups.

Method	Area	*n*	Mean	Standard Deviation	Minimum	Maximum
Micro-CT	Vestibular	60	0.101	0.107	0.003	0.400
	Oral	60	0.077	0.063	0.004	0.230
	Pulpal	60	0.104	0.079	0.011	0.310
Optical Microscopy	Vestibular	60	0.071	0.055	0.004	0.240
	Oral	60	0.094	0.065	0.005	0.240
	Pulpal	60	0.100	0.080	0.004	0.310

**Table 3:** Comparison between microscopy and micro-CT results using the Wilcoxon Signed Ranks Test.

Area	Ranks	*n*	Mean Rank	Sum of Ranks	*p* ^sig^
Vestibular	Negative Ranks	26	39.02	1014.5	0.218 ^is^
	Positive Ranks	32	21.77	696.5	
Oral	Negative Ranks	16	25.28	404.5	<0.001 ^s^
	Positive Ranks	44	32.40	1425.5	
Pulpal	Negative Ranks	25	35.54	888.5	0.845 ^is^
	Positive Ranks	35	26.90	941.5	

Notations: *p* ^sig^, significance level; ^s^, statistically significant; ^is^, statistically insignificant.

**Table 4:** Comparison between the adhesive layer widths *w* obtained with micro-CT for Groups 1 and 2.

Area	Group	*n*	Mean ± SD	Standard Error Mean	Mean Rank	Sum of Ranks	*p* ^sig^
Vestibular	1	30	0.136 ± 0.096	0.017	32.32	969.5	0.420 ^is^
	2	30	0.123 ± 0.102	0.019	28.68	860.5	
Oral	1	30	0.139 ± 0.068	0.012	34.32	1029.5	0.090 ^is^
	2	30	0.110 ± 0.053	0.010	26.68	800.5	
Pulpal	1	30	0.138 ± 0.065	0.012	31.78	953.5	0.569 ^is^
	2	30	0.129 ± 0.086	0.016	29.22	876.5	

Notations: *p* ^sig^, significance level; ^is^, statistically insignificant.

**Table 5:** Comparison between the adhesive layer widths *w* obtained with micro-CT for Groups 1 and 3.

Area	Group	*n*	Mean ± SD	Standard Error Mean	Mean Rank	Sum of Ranks	*p* ^sig^
Vestibular	1	30	0.136 ± 0.096	0.017	38.10	1143.00	0.001 ^s^
	3	30	0.062 ± 0.044	0.008	22.90	687.00	
Oral	1	30	0.139 ± 0.068	0.012	39.33	1180.00	<0.001 ^s^
	3	30	0.068 ± 0.045	0.008	21.67	650.00	
Pulpal	1	30	0.138 ± 0.065	0.012	33.55	1006.50	0.176 ^is^
	3	30	0.117 ± 0.075	0.014	27.45	823.50	

Notations: *p* ^sig^, significance level; ^s^, statistically significant; ^is^, statistically insignificant.

**Table 6:** Comparison between the adhesive layer widths *w* obtained with micro-CT for Groups 3 and 4.

Area	Group	*n*	Mean ± SD	Standard Error Mean	Mean Rank	Sum of Ranks	*p* ^sig^
Vestibular	3	30	0.062 ± 0.044	0.008	39.87	1196.0	<0.001 ^s^
	4	30	0.024 ± 0.008	0.002	21.13	634.0	
Oral	3	30	0.068 ± 0.045	0.008	41.02	1230.5	<0.001 ^s^
	4	30	0.026 ± 0.010	0.002	19.98	599.5	
Pulpal	3	30	0.117 ± 0.075	0.014	43.67	1310.0	<0.001 ^s^
	4	30	0.024 ± 0.007	0.001	17.33	520.0	

Notations: *p* ^sig^, significance level; ^s^, statistically significant.

**Table 7:** Modelling of the functions of surface area versus width/thickness of the adhesive—Groups 1 and 2.

Group	Parameters w (mm); S (mm^2^)	Area *S* of the Surface of Adhesive Layer with a Certain Width *w*		
		$\left[w_{0},(w_{0}+w_{M}\right)/2)$	$\left[\left(w_{0}+w_{M}\right)/2,w_{M}\right)$	$\left[w_{M},w_{max}\right)$
1	*w*_0_ = 0;	$f\left(w\right)=40w^{2}$ f′(w)=80	$g\left(w\right)=-5.714w^{2}+0.8w-0.014$ g′(w)=−11.428w+0.8	$h\left(w\right)=0.034w^{2}-0.045w+0.017$ h′(w)=0.068w−0.045
	*S*_0_ = 0			
	*w*_M_ = 0.07			
	*S*_M_ = 0.014			
	*w*_I_ = 0.2			
	*S*_I_ = 0.008			
	*w*_max_ = 0.4			
	*S*_m_ = 0.002	$Volume\;of\;the\;adhesive:\;V_{1}=737.13\times 10^{-5}\;{\mathrm{mm}}^{3}$		
2	*w*_0_ = 0.02;	$f\left(w\right)=4.375w^{2}-0.175w-0.088$ f′(w)=8.75w−0.175	$g\left(w\right)=-4.375w^{2}-0.875w-0.012$ $g^{\prime }\left(w\right)=-8.75w-0.875$	$h\left(w\right)=0.1w^{2}-0.09w-0.004$ h′(w)=0.2w−0.09
	*S*_0_ = 0			
	*w*_M_ = 0.1			
	*S*_M_ = 0.014			
	*w*_I_ = 0.2			
	*S*_I_ = 0.008			
	*w*_max_ = 0.4			
	*S*_m_ = 0.002	$Volume\;of\;the\;adhesive:\;V_{2}=481.5\times 10^{-5}\;{\mathrm{mm}}^{3}$		

**Table 8:** Modelling of the functions of surface area versus width/thickness of the adhesive—Groups 3 and 4.

Group	Parameters w (mm); S (mm^2^)	Area *S* of the Surface of Adhesive Layer with a Certain Width *w*		
		$\left[0,w_{M}\right)$	$\left[w_{M},w_{I}\right)$	$\left[w_{I},w_{max}\right)$
3	*w*_M_ = 0.02	$f\left(w\right)=40w^{2}$ f′(x)=80w	$g\left(w\right)=2.188w^{2}-0.438w+0.04$ $g^{\prime }\left(w\right)=4.376w-0.438$	h(w)=−0.0033w+0.0023 h′(w)=−0.0033
	*S*_M_ = 0.016			
	*w*_I_ = 0.1			
	*S*_I_ = 0.002			
	*w*_max_ = 0.4			
	*S*_m_ = 0.001	$Volume\;of\;the\;adhesive:\;V_{3}=322.8\times 10^{-5}\;{\mathrm{mm}}^{3}$		
4	*w*_M_ = 0.015	$f\left(w\right)=66.67w^{2}$ f′(x)=133.34w	$g\left(w\right)=3.077w^{2}+0.492w-0.022$ g′(w)=6.154w+0.492	h(w)=−0.0071w+0.0026 h′(w)=−0.0071
	*S*_M_ = 0.015			
	*w*_I_ = 0.18			
	*S*_I_ = 0.002			
	*w*_max_ = 0.36			
	*S*_m_ = 0	$Volume\;of\;the\;adhesive:\;V_{4}=217\times 10^{-5}\;{\mathrm{mm}}^{3}$		

## Appendix A

Ascertainment of the functions of areas of the surfaces of adhesives regarding their widths–for Groups 1 and 2

Parabolic functions are considered for each portion of the *S*(*w*) graphs in Figure 10(a1).

For the first portion, for $x\in \left[w_{0},(w_{M}+w_{0})/2\right)$:

(A1) $$f\left(w\right)=aw^{2}+bw+c\;\mathrm{and}\;f\prime \left(w\right)=2aw+b,\;\mathrm{where}\;a,b,c=cst.$$

The constants *a*, *b* and *c* are obtained by imposing the conditions extracted from Figure 10(a1):

(A2) $$f\left(w_{0}\right)=0;\;f\left[\left(w_{M}+w_{0}\right)/2\right]=S_{M}/2;\;df/dw\left(w_{0}\right)=0.$$

With Equation (A1) in (A2), the coefficients are:

(A3) $$a_{1}=2S_{M}/{\left(w_{M}-w_{0}\right)}^{2};\;b_{1}=-4w_{0}S_{M}/{\left(w_{M}-w_{0}\right)}^{2};\;c_{1}=w_{0}^{2}S_{M}/{\left(w_{M}-w_{0}\right)}^{2}.$$

Using the parameters in Table 7, one thus obtains from Equation (A3) the coefficients *a*, *b* and *c* for Groups 1 and 2: for Group 1, *a*_1_ = 40 and *b*_1_ = *c*_1_ = 0, while for Group 2, *a*_1_ = 4.375, *b*_1_ = −0.175 mm and *c*_1_ = 0.0875 mm^2^.

For the second portion of the *S*(*w*) graph, i.e., for $x\in \left[(w_{M}+w_{0})/2,w_{M}\right)$, another parabolic function, *g*(*w*) is considered, with the following conditions extracted from Figure 10(a1):

(A4) $$g\left(w_{M}\right)=S_{M};\;g\left[\left(w_{M}+w_{0}\right)/2\right]=S_{M}/2;\;dg/dw\left(\left(w_{M}+w_{0}\right)/2\right)=2S_{M}/\left(w_{M}-w_{0}\right).$$

With Equation (A4) in (A1),

(A5) $$a_{2}=-2S_{M}/{\left(w_{M}-w_{0}\right)}^{2};\;\;b_{2}=4w_{M}S_{M}/{\left(w_{M}-w_{0}\right)}^{2};\;\;c_{2}=S_{M}-2w_{M}^{2}S_{M}/{\left(w_{M}-w_{0}\right)}^{2}.$$

Using the parameters in Table 7, one thus obtains from Equation (A5) the coefficients *a*, *b* and *c* for Groups 1 and 2: for Group 1, *a*_2_ = −40, *b*_2_ = 0.8 mm and *c*_2_ = −0.014 mm^2^, while for Group 2, *a*_2_ = −4.375, *b*_2_ = 0.175 mm and *c*_2_ = 0.012 mm^2^.

For the third portion of the *S*(*x*) graph, i.e., for $w\in \left[(w_{M},w_{max}\right)$, a third parabolic function, *h*(*w*) is considered, with the following conditions extracted from Figure 10(a1):

(A6) $$h\left(w_{M}\right)=S_{M};\;h\left(w_{I}\right)=S_{I};\;h\left(w_{max}\right)=S_{m}.$$

With Equation (A6) in (A1),

(A7) $$a_{3}=\frac{S_{M}\left(w_{max}-w_{I}\right)+S_{m}\left(w_{I}-w_{M}\right)-S_{I}\left(w_{max}-w_{M}\right)}{w_{max}w_{I}\left(w_{max}-w_{I}\right)+w_{I}w_{M}\left(w_{I}-w_{M}\right)-w_{max}w_{M}\left(w_{max}-w_{M}\right)}\;b_{3}=-\frac{S_{M}\left(w_{max}^{2}-w_{I}^{2}\right)+S_{m}\left(w_{I}^{2}-w_{M}^{2}\right)-S_{I}\left(w_{max}^{2}-w_{M}^{2}\right)}{w_{max}w_{I}\left(w_{max}-w_{I}\right)+w_{I}w_{M}\left(w_{I}-w_{M}\right)-w_{max}w_{M}\left(w_{max}-w_{M}\right)}\;;\;c_{3}=S_{M}-a_{3}w_{M}^{2}-b_{3}w_{M}.$$

Using the parameters in Table 7, one thus obtains from Equation (A7) the coefficients *a*, *b* and *c* for Groups 1 and 2: for Group 1, *a*_3_ = −0.034, *b*_3_ = −0.045 mm and *c*_3_ = −0.017 mm^2^, while for Group 2, *a*_3_ = 0.1, *b*_3_ = −0.09 mm and *c*_3_ = 0.004 mm^2^.

The functions *f*(*w*), *g*(*w*) and *h*(*w*) of the *S*(*w*) curve are provided in Table 7, as well as their gradients *f*’(*w*), *g*’(*w*) and *h*’(*w*)—which are also shown in Figure 10(a2).

## Appendix B

### Ascertainment of the Functions of Areas of the Surfaces of Adhesives Regarding Their Widths–for Groups 3 and 4

Parabolic functions are also considered for each portion of the *S*(*w*) graph in Figure 10(b1).

For the first portion, for $x\in \left[0,w_{M}\right)$, with a function similar to the one in Equation (A1), the constants *a*, *b* and *c* are obtained by imposing the conditions extracted from Figure 10(b1):

(A8) $$f\left(0\right)=0;\;f\left[w_{M}\right]=S_{M};\;df/dw\left(0\right)=0.$$

With Equation (A1) in (A8), the three coefficients are:

(A9) $$a_{1}=S_{M}/w_{M}^{2};\;b_{1}=c_{1}=0$$

Using the parameters in Table 8, one thus obtains from Equation (A9), for Group 3, *a*_1_ = 40, while for Group 4, *a*_1_ = 66.67.

For the second portion of the *S*(*w*) graph, i.e., for $x\in \left[w_{M},w_{I}\right)$, another parabolic function, *g*(*w*) is considered, with the following conditions extracted from Figure 10(b1):

(A10) $$g\left(w_{M}\right)=S_{M};\;g\left[w_{I}\right]=S_{I};\;dg/dw\left(w_{I}\right)=0.$$

With Equation (A1) in (A10),

(A11) $$a_{2}=(S_{M}-S_{I})/{\left(w_{M}-w_{I}\right)}^{2};\;b_{2}=-2w_{I}(S_{M}-S_{I})/{\left(w_{M}-w_{I}\right)}^{2};\;c_{2}=[S_{M}w_{I}^{2}-S_{M}w_{M}\left(2w_{I}-w_{M}\right)]/{\left(w_{M}-w_{I}\right)}^{2}.$$

Using the parameters in Table 8, one thus obtains from Equation (A11) the coefficients *a*, *b* and *c* for Groups 3 and 4: for Group 3, *a*_2_ = 2.188, *b*_2_ = 0.438 mm and *c*_2_ = 0.024 mm^2^, while for Group 4, *a*_2_ = 3.077, *b*_2_ = −0.492 mm and *c*_2_ = 0.022 mm^2^.

For the third portion of the *S*(*x*) graph, i.e., for $w\in \left[(w_{I},w_{max}\right)$, a linear function, *h*(*w*) is considered,

(A12) $$h\left(w\right)=d_{3}w+e_{3};\;h\prime \left(w\right)=d_{3}.$$

with the following conditions extracted from Figure 10(b1):

(A13) $$h\left(w_{I}\right)=S_{I};\;h\left(w_{max}\right)=S_{m}.$$

With Equation (A13) in (A12),

(A14) $$d_{3}=-\left(S_{I}-S_{m}\right)/\left(w_{max}-w_{I}\right);\;e_{3}=\left(S_{I}w_{max}-S_{m}w_{I}\right)/\left(w_{max}-w_{I}\right).$$

Using the parameters in Table 8, one thus obtains from Equation (A14) the coefficients: for Group 3, *d*_3_ = −0.0033 mm and *e*_3_ = 0.0023 mm^2^, while for Group 4, *d*_3_ = −0.0071 mm and *e*_3_ = 0.0026 mm^2^.

The functions *f*(*w*), *g*(*w*) and *h*(*w*) of the *S*(*w*) curve are provided in Table 8, as well as their gradients *f*’(*w*), *g*’(*w*) and *h*’(*w*)—which are also shown in Figure 10(b2).
